# Cases of Phagedenic Ulceration

**Published:** 1828-01

**Authors:** 

**Affiliations:** St. Bartholomew's Hospital.


### PHAGEDENIC ULCERATION.

*Cases of Phagedenic Ulceration.* Treated by Mr. Earle, at St. Bartholomew's Hospital.

Eliza Marshall, æt. twenty-nine, admitted into Patience' ward, under Mr. Earle, November 16th.

This is the third time of her admission into the hospital for the same affection: the first was about two years ago, when she was speedily cured by the use of the strong Nitric Acid; and the same treatment was successfully repeated upon the breaking out of the wound last spring. Each time the sore has occupied its present situation, which is on the left buttock, between the anus and the pudendum. She always enjoyed particularly good health, with these exceptions, never having been subject to any other venereal affection, except one small sore, some years ago, which was very soon healed, and is not aware that she ever took mercury.

About a fortnight ago she began to feel unwell, but nothing appeared till Sunday, November 12th, when she perceived a small lump on the old spot, which in a few hours burst into a wound, that has continued increasing to the present time, but there has been no running or sore. It is now about three inches in diameter, with irregular and jagged edges, having the dark, speckled, and dirty appearance characteristic of this peculiar form of venereal disease. There is a foul discharge, accompanied with much pain and considerable fetor. She has also a phagedenic ulceration, about the size of half-a-crown, on the left leg, which has existed two months. She complains of headache and thirst; bowels confined; pulse 110. The Nitric Acid was freely applied, after which she soon became much easier.

**Dry lint to the wound.—Tinct. Opii ʒj statim.—Mist. Salin. quartis horis.—Liq. Ant. Tart. m. xx. in sing. dos.—Fumigation to the leg.**

17th.—The ulcerative process has stopped, and the constitutional irritation much abated.

**Contr ut antea.**

18th.—A black eschar fills up the wound, which has not, however, increased. General health improved. The wound in the leg looks better.

**Lotio Opii cum Acid. Nit. bis indies.**

19th.—Better. Eschar not yet come away.—Continue.

The patient had nearly recovered of the first ulcer, when, on the 7th of December, another one, of the same nature, broke out on the right labium, accompanied with great swelling of the neighbouring parts: it began with a small pimple, and in two days the sore was as large as a half-crown piece. The Nitric Acid was applied to it on the 9th instant, but the diseased process not having been arrested, the acid was reapplied the next day, with decided success. The sloughs came away, and the sore is now nearly healed. But, within the last week, the old sore has again fallen almost into its former condition. Her general health is apparently very good, and has been throughout; but there is a strange disposition to phagedenic ulceration, without ever having had any primary sore of that description, (at least as far as her statement can be credited;) showing the disease to be purely constitutional with her, which is, in fact, in direct opposition to Carmichael's doctrine.

Case II.—Sarah Mitcham, æt. eighteen, admitted into Patience' ward, under Mr. Earle, October 27th. She gives the following account of herself:—That, about five weeks since, several hard whitish bumps appeared on the pudendum, which were attended with an itching sensation and some heat when she was warm. She had previously much scalding in making water, but she did not observe any discharge till after these tumors appeared, when a very profuse one came on. She for some time neglected these symptoms; but, about a fortnight ago, a small pimple came out on the left side of the labium, which soon broke into a painful sore, it was not however till this had attained the size of a shilling that she applied to a surgeon, who gave her some black wash, which she used with much advantage till poverty obliged her to leave it off; upon which the sore immediately began to spread again. Since she discontinued the wash, she has applied nothing but bread and beer-ground poultice.

The sore is at present of a most formidable nature: it extends for nearly a hand's breadth on each side of the labia upon either thigh, and goes backwards on the buttock and perineum, having a dark brown and ragged appearance, with an intolerable fetor; the edges are uneven, and violently inflamed; the labia are completely eroded, but the raphe of the perineum seems uninjured; there is a copious discharge from the vagina. The constitution is apparently but little affected: pulse 100, yet not full; bowels in good order, and no fever. The strong Nitric Acid was freely applied for some time, during which she suffered great agony. The surface was then dressed with dry lint.

Ol. Ricini ℥j.; Tinct. Opii gtt. L.

In about an hour she became perfectly easy, and went to sleep.

28th.—The phagedenic process has apparently stopped, except on the left side, to which the Nitric Acid was again applied.

Tinct. Opii gtt. xl.

The pain of this operation did not cease till evening, when she took Pulv. Ipecac. c. gr. x.

29th.—Better. The whole surface is now destroyed, and the edges clean. Sore to be washed with Sodæ Chlor. ʒij. Aquæ Oj. several times in the day. General health continues good.

30th.—Part of the eschar has come off with the dressing.

Quinin. Sulph. gr ij.; Infus. Menthæ Sulph. ℥j. fiat haust. bis die sumend.—Wound dressed with Lotio Opii ℥j.; Acid. Nit. gtt. iij.

31st.—Much the same.

Edges smeared with Ung. Cetacei et Cerat. Res. Flav. āā ℥j.—Lotion as before.

November 1st.—The eschar is entirely removed, and the sore looks clean and well.

5th.—Granulations are sprouting fast, and the edges are skinning over. The vaginal discharge has much diminished, and great care is taken to keep the parts clean.

10th.—Rapidly improving; the wound is much contracted.

Contr ut antea.

15th.—She is getting fast well; the wound is very much diminished.

She left the hospital cured, soon after.

Case III.—Mary Ann Godfrey, æt. twenty-two, admitted into Patience' ward, under Mr. Earle, November 16th. States that, about a fortnight ago, the labia on both sides began to swell to a considerable size, but there was neither running nor sore. In the course of two or three days, a small pimple appeared on the left labium, which quickly became a sore, increasing in size till her admission, but for which she had done nothing. Her general health has always been very good; she has lived in an open and airy situation for some time.

The sore has now destroyed the left labium almost entirely, and is fast encroaching on the opposite one. The clitoris appears to be included in the ulcer, which reaches downwards to the fourchette. The surface is deep and irregular, covered with foul sloughs, and the margin is red and inflamed. She suffers much pain, and her constitution is greatly disordered; tongue foul; pulse 115; bowels confined; skin hot and dry; and she has much thirst.

Applic^r Acid. Nit. fort, et postea Tinct. Opii m. xl. Pulv. Ipecac. comp. gr. x. h. s. Dry lint to the wound.

17th.—Somewhat better today, but the sloughing is not quite stopped at the upper part of the wound, which is in other respects much the same; bowels freely opened.

Repet^r Acid. Nit. fort. et postea Tinct. Opii m. xxx. Pulv. Ipecac. comp. h. s.

18th.—The sloughing still continues in the upper part of the wound, and there is much pain in the part; pulse 100, and full; tongue furred.

Applic^r Cucurbit. Crunt. ad ℥xij. lumbis.—Continue dry lint to the wound.

19th.—Much the same.

The Protonitrate of Mercury to be applied.

Two days after the application of the Protonitrate, the sloughs came off, leaving a healthy granulating surface, which has been gradually healing since, without further interruption; and she soon left the hospital perfectly cured.

Although the improvement in this case seems chiefly to be attributed to the nitrate of mercury, it is questionable whether it did not depend as much on the strength of the nitric acid forming the protonitrate being greater than that of the pure acid which had been previously applied; for there is certainly great variety in this article, at least as it is manufactured in this hospital.

---

